# Plasma brain natriuretic peptide as a surrogate marker for cardioembolic stroke

**DOI:** 10.1186/1471-2377-8-45

**Published:** 2008-12-11

**Authors:** Kazushi Yukiiri, Naohisa Hosomi, Takayuki Naya, Tsutomu Takahashi, Hiroyuki Ohkita, Mao Mukai, Hisashi Masugata, Koji Murao, Masaki Ueno, Takehiro Nakamura, Hiroaki Dobashi, Takanori Miki, Yasuhiro Kuroda, Masakazu Kohno

**Affiliations:** 1 Department of Cardiorenal and Cerebrovascular Medicine, Division of Stroke, Kagawa University School of Medicine, 1750-1 Ikenobe, Miki-cho, Kagawa 761-0793, Japan

## Abstract

**Background:**

Cardioembolic stroke generally results in more severe disability, since it typically has a larger ischemic area than the other types of ischemic stroke. However, it is difficult to differentiate cardioembolic stroke from non-cardioembolic stroke (atherothrombotic stroke and lacunar stroke). In this study, we evaluated the levels of plasma brain natriuretic peptide in acute ischemic stroke patients with cardioembolic stroke or non-cardioembolic stroke, and assessed the prediction factors of plasma brain natriuretic peptide and whether we could differentiate between stroke subtypes on the basis of plasma brain natriuretic peptide concentrations in addition to patient's clinical variables.

**Methods:**

Our patient cohort consisted of 131 consecutive patients with acute cerebral infarction who were admitted to Kagawa University School of Medicine Hospital from January 1, 2005 to December 31, 2007. The mean age of patients (43 females, 88 males) was 69.6 ± 10.1 years. Sixty-two patients had cardioembolic stroke; the remaining 69 patients had non-cardioembolic stroke (including atherothrombotic stroke, lacunar stroke, or the other). Clinical variables and the plasma brain natriuretic peptide were evaluated in all patients.

**Results:**

Plasma brain natriuretic peptide was linearly associated with atrial fibrillation, heart failure, chronic renal failure, and left atrial diameter, independently (F_4,126 _= 27.6, p < 0.0001; adjusted R^2 ^= 0.45). Furthermore, atrial fibrillation, mitral regurgitation, plasma brain natriuretic peptide (> 77 pg/ml), and left atrial diameter (> 36 mm) were statistically significant independent predictors of cardioembolic stroke in the multivariable setting (Χ^2 ^= 127.5, p < 0.001).

**Conclusion:**

It was suggested that cardioembolic stroke was strongly predicted with atrial fibrillation and plasma brain natriuretic peptide. Plasma brain natriuretic peptide can be a surrogate marker for cardioembolic stroke.

## Background

Stroke is a major cause of serious long-term disability and death [1]. Acute ischemic stroke accounts for approximately 70% of all strokes, and is caused by embolic or atherosclerotic occlusion in the cerebral vessels. Furthermore, cardioembolic stroke generally results in more severe disability, since it typically has a larger ischemic area than the other types of ischemic stroke.

However, it is difficult to diagnose the ischemic stroke subtypes exactly at admission. And, it takes 2 weeks to identify paroxysmal atrial fibrillation (Af) since standard electrocardiography (ECG), 24 hour ECG recording, and 14-days ambulatory ECG monitoring is necessary [2]. Furthermore, there are the patients with patent foramen ovale (PFO), pulmonary arteriovenous fistula, or the others that are the causes of cardioembolic stroke and show basically normal finding in ECG and transthoracic echocardiogram (TTE). It nevertheless remains important to differentiate cardioembolic stroke from non-cardioembolic stroke (atherothrombotic stroke and lacunar stroke), since acute and secondary prevention treatment differ in these cases: in cardioembolic stroke, anti-coagulant agents (e.g. warfarin) are most effective for secondary prevention, whereas in non-cardioembolic stroke, anti-platelet agents (e.g. aspirin, cilostazol, or clopidogrel) are commonly used in Japan.

Plasma brain natriuretic peptide (BNP) concentration has seen widespread use in Japan as a biochemical marker of heart failure [3]. In the same time, plasma BNP is known to increase in patients with Af [4], cardiomyopathy [5], heart failure [6], myocardial infarction [7], or chronic renal failure [8]; but little is known about plasma BNP in ischemic stroke. In this study, plasma BNP was evaluated in a cohort of acute ischemic stroke patients, with the goal of assessing whether one could differentiate cardioembolic stroke from non-cardioembolic stroke with non-invasive examination.

## Methods

### Subjects

One hundred thirty one consecutive patients with acute ischemic stroke were admitted to Kagawa University School of Medicine Hospital within 24 hours after their stroke onset from January 1, 2005 to December 31, 2007. In these patients, there were 25 patients who variously had heart failure (n = 14; serologic data of left ventricular ejection fraction < 40%), cardiomyopathy (n = 8; past diagnosis with echocardiography and coronary angiography), old myocardial infarction (n = 11; suggestive ECG changes and clear evidence of asynergy with echocardiography) or chronic renal failure (n = 12; serologic data of blood urea nitrogen > 30 mg/dl, creatinine > 1.5 mg/dl, or creatinine clearance < 70 ml/min) all of which were known to increase plasma BNP.

Plasma BNP was evaluated at the first morning after admission in all 131 patients. In the present study, Af was diagnosed as permanent Af or paroxysmal Af, with standard ECG, 24-hour ECG recording, and 14-day ambulatory ECG monitoring [2]. Patients were evaluated national institute of health stroke scale (NIHSS) score at their admission, and were diagnosed as having cardioembolic stroke or non-cardioembolic stroke (including atherothrombotic stroke, lacunar stroke, or the other), using echocardiography, brain computed tomography, magnetic resonance imaging, magnetic resonance angiography, and carotid ultrasonography by two stroke specialists (N.H., T.T.). Final diagnosis of stroke subtype was made at the patients' discharge based on Trial of Org 10172 in Acute Stroke Treatment (TOAST) criteria [9]. We blinded the BNP value to the physicians who diagnosed the stroke subtypes. Informed consent was obtained from all patients; and, this study was approved by the investigational review board of the Kagawa University School of Medicine.

### Echocardiography

TTE was performed in all patients, and left ventricular (LV) ejection fraction was calculated as (1 – end-systolic volume/end-diastolic volume) × 100. Chamber size was measured by the modified Simpson's method on 2-dimensional echocardiography [10]. LV end-diastolic dimension was measured on M-mode echocardiography [11]. Transesophageal echocardiography (TEE) was performed to measure left atrial appendage (LAA) flow and detect PFO and shunt flow from right to left atrium.

### Plasma Brain Natriuretic Peptide (BNP)

Blood samples were taken on the first morning after admission. Blood was immediately separated into chilled tubes containing potassium ethylenediaminetetraacetic acid and centrifuged for 30 minutes. Plasma BNP concentrations were then determined with a specific immunoradiometric assay (Shionoria BNP Kit, Shionogi Co., Ltd., Tokyo, Japan) [12]. Our normal range of BNP is less than 18.4 pg/ml.

### Statistical analysis

Summary statistics were expressed as mean ± standard deviation or medians (ranges of 25 to 75 percentile) for continuous variables, and frequencies and percentages for discrete variables. To estimate the reproducibility of measurements of dimension and flow velocity with echocardiography, the echocardiography of 10 volunteers were evaluated by 8 trained cardiologists who had evaluated echocardiography in this study. Intraobserver coefficients of variation of dimension and flow velocity of echocardiography were 7.0 and 9.2%, respectively, and interobserver coefficients of variation of dimension and area of echocardiography were 9.6 and 10.8%, respectively. Comparisons of clinical characteristics and ultrasonographic measurements between the stroke subtypes were made with either one way-ANOVA with Bonferroni's correction for multiple comparisons (for continuous variables) or Fisher exact tests (for discrete variables). Differences in NIHSS score among groups were examined using Kruskal-Wallis test. Multiple linear regression analysis was used to assess whether plasma BNP could be adequately predicted from clinical variables. Our regression strategy was to undertake all subsets regressions, with no forced independent variables. The regression models were determined from this procedure, and represented the best (in terms of maximal R^2^) of all possible regression models. Each of the independent variables in the regression models was nominally significant, with p < 0.05. Multivariable logistic regression was utilized to assess the relative importance of variables that was found an association with cardioembolic stroke in initial univariate analyses.

## Results

Our study cohort consisted of 131 patients: 62 cardioembolic stroke patients and 69 patients with non-cardioembolic stroke (24 atherothrombotic stroke, 21 lacunar stroke, and 24 undetermined subtype of ischemic stroke). All of the paroxysmal Af patients were in sinus rhythm at admission. In these patients, there were 25 patients who variously had heart failure (n = 14), cardiomyopathy (n = 8), old myocardial infarction (n = 11), or chronic renal failure (n = 12). Clinical characteristics of the patients were given in Table 1. There were no significant differences between cardioembolic stroke and non-cardioembolic stroke in these parameters, with the exceptions of plasma BNP and distribution of Af and mitral regurgitation: plasma BNP was significantly increased in the cardioembolic stroke patients compared with the non-cardioembolic stroke patients, and Af was highly distributed in cardioembolic stroke (p < 0.001; respectively).

**Table 1:** Clinical characteristics and cardiac function ultrasonographic variables.

Variable	Non-Cardioembolic Stroke (n = 69)	Cardioembolic Stroke (n = 62)
Age (years)	69.9 ± 9.9	69.8 ± 10.4
Sex, F/M	19/50	24/38
Systolic Blood Pressure (mmHg)	177 ± 11	182 ± 14
Body Mass Index, kg/m^2^	23.6 ± 2.5	24.1 ± 3.4
Blood Urea Nitrogen, mg/dl	15.2 ± 5.6	13.7 ± 4.3
Creatinine, mg/dl	1.10 ± 0.27	1.06 ± 0.37
Hypertension, yes/no (%)	46/23 (66.7%)	41/21 (66.1%)
Dislipidemia, yes/no (%)	15/54 (21.7%)	16/46 (25.8%)
Diabetes Mellitus, yes/no (%)	17/52 (24.6%)	18/44 (29.0%)
Smoking, yes/no (%)	42/27 (60.9%)	34/28 (54.8%)
Atrial fibrillation (including permanent and paroxysmal Af), yes/no (%)	2/67 (2.9%)	49/13 (79.0%)**
Mitral stenosis, yes/no (%)	0/69 (0%)	3/59 (4.8%)
Mitral regurgitation, yes/no (%)	20/49 (29.0%)	34/28 (54.8%)*
Heart Failure, yes/no (%)	5/64 (7.2%)	9/53 (14.5%)
Old Myocardial Infarction, yes/no (%)	3/66 (4.3%)	8/54 (12.9%)
Cardiomyopathy, yes/no (%)	4/65 (5.8%)	4/58 (6.5%)
Chronic Renal Failure, yes/no (%)	7/62 (10.1%)	5/57 (8.1%)
Plasma BNP (pg/ml)	49.6 ± 43.3	106.6 ± 31.5**
Duration after ischemic onset to BNP measurement (hours)	9.2 ± 4.2	9.6 ± 3.8
NIH Stroke Scale score at admission: median (25%–75%)	11 (9–14)	12 (9–18)
Beta-blocker, yes/no (%)	12/57 (17.4%)	13/49 (21.0%)
Angiotensin receptor blocker, yes/no (%)	36/33 (52.2%)	25/37 (40.3%)
Angiotensin converting enzyme inhibitor, yes/no (%)	7/62 (10.1%)	9/53 (14.5%)
E/A	0.8 ± 0.2	1.7 ± 0.6**
LA diameter (mm)	33.9 ± 5.4	41.6 ± 6.8**
LV end-diastolic diameter (mm)	47.9 ± 2.2	47.8 ± 2.2
LV end-systolic diameter (mm)	28.9 ± 2.3	29.1 ± 2.3
Interventricular septum thickness (mm)	12.2 ± 2.8	12.7 ± 2.8
LV posterior wall thickness (mm)	12.3 ± 2.4	12.8 ± 2.5
LV ejection fraction (%)	62.1 ± 10.0	60.8 ± 13.3
LAA flow (cm/s)	76.6 ± 14.1	35.0 ± 22.0**

LA; left atrium. LV; left ventricular. LAA; left atrial appendage.

*: p < 0.01, **: p < 0.001 compared with patients in non-cardioembolic stroke.

The values in this table: means and standard deviations or medians (ranges of 25 to 75 percentile) are given for the continuous variables; numbers of patients are given for the categorical variables. %: percentile.

Ultrasonographic variables for cardiac function with TTE are given in Table 1. E/A and left atrial (LA) diameter were significantly increased, and LAA flow was significantly decreased, in cardioembolic stroke compared with non-cardioembolic stroke (p < 0.001; respectively; E/A was not available for the permanent Af patients). There were no significant differences between the two subgroups in the other cardiac function parameters obtained with ultrasonographic examination.

To define the factors that influenced on the plasma BNP, we screened through the factors in Table 1. Plasma BNP was significantly associated with blood urea nitrogen, creatinine, Af, heart failure, old myocardial infarction, cardiomyopathy, chronic renal failure, E/A, LA diameter, LAA flow, and LV ejection fraction. To assess the relative importance of those factors on plasma BNP, we utilized ordinal multivariate linear regressions. Among them, we found that Af, heart failure, chronic renal failure, and LA diameter were statistically significant predictors of plasma BNP in the multivariable setting (F_4,126 _= 27.6, p < 0.0001; adjusted R^2 ^= 0.45). Statistical results of multivariate linear regression are given in Table 2. In other words, blood urea nitrogen, creatinine, old myocardial infarction, cardiomyopathy, E/A, LAA flow, and LV ejection fraction turned out not to be associated with plasma BNP after controlling Af, heart failure, chronic renal failure, and LA diameter in acute stroke patients. None of stroke size, NIHSS score, and the duration between plasma BNP measurement from the stroke onset was associated with plasma BNP concentration.

**Table 2:** Least squares linear regression of plasma BNP

Variable	Coefficient	Std Error	T	P
Constant	-3.175	17.016	-0.187	0.8523
Atrial Fibrillation	46.953	7.214	6.509	< 0.0001
Heart Failure	50.057	11.538	4.338	< 0.0001
Chronic Renal Failure	36.802	11.696	3.147	0.0021
LA dimension	1.404	0.475	2.957	0.0037

In the regression equation, atrial fibrillation, heart failure, and chronic renal failure are indicator variables, coded 1 for subjects with past or present disease and 0 without disease. Units of the other variables are as in Table 1. Summary statistics relating to the regression fit include ANOVA (F_4,126 _= 27.6, p < 0.0001; adjusted R^2 ^= 0.45). Plasma BNP was associated with atrial fibrillation, heart failure, chronic renal failure, and LA dimension, independently.

**Figure 1:**
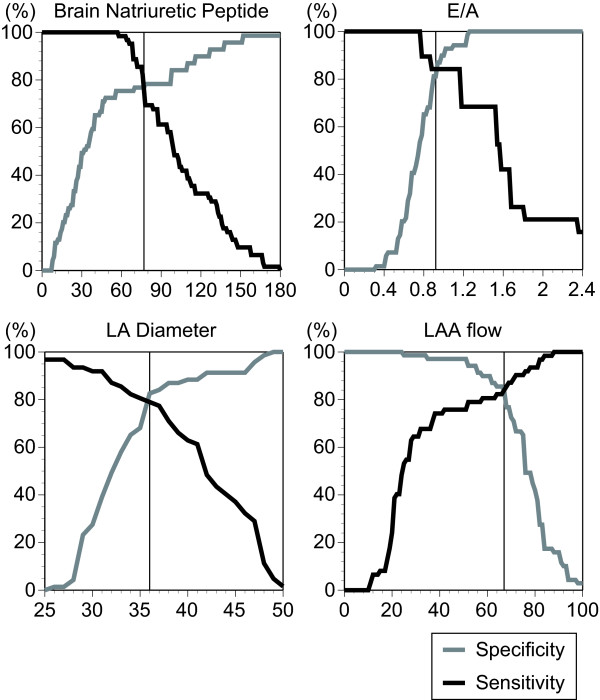
Sensitivity (black line) and specificity (gray line) of plasma BNP, E/A, LA diameter, and LAA flow for classifying cardioembolic stroke from non-cardioembolic stroke.

To identify the cardioembolic stroke from non-cardioembolic stroke, we have defined the sensitivity and specificity of plasma BNP, E/A, LA diameter, and LAA flow that had significant difference between stroke subtypes. E/A was not available for the permanent Af patients. The sensitivity and specificity of plasma BNP, E/A, LA diameter, and LAA flow on classifying cardioembolic stroke from non-cardioembolic stroke were shown in Figure 1. In Table 3, we gave cutoffs at the crossed point of sensitivity and specificity for each of plasma BNP, E/A, LA diameter, and LAA flow, as well as prediction probabilities, for classifying cardioembolic stroke from non-cardioembolic stroke. E/A (> 0.92), LA diameter (> 36 mm), and LAA flow (> 67 cm/s) achieved high discrimination between the two stroke subtypes. Those factors had more than 80% overall accuracy on diagnosing cardioembolic stroke from non-cardioembolic stroke. Plasma BNP (> 77 pg/ml) had slightly weaker predictive power than the others. However, it also had 76.3% overall accuracy.

**Table 3:** Cut off points and prediction probabilities in the stroke.

Variable	Cut off	CE stroke	NCE stroke	Overall Accuracy
Plasma BNP	77 pg/ml	47/62 (75.8%)	53/69 (76.8%)	100/131 (76.3%)
E/A	0.92	16/19 (84.2%)	56/69 (81.2%)	72/88 (81.8%)
LA diameter	36 mm	49/62 (79.0%)	57/69 (82.6%)	106/131 (80.9%)
LAA flow	67 cm/s	52/62 (83.9%)	58/69 (84.1%)	110/131 (84.0%)

CE: cardioembolic; NCE non-cardioembolic.

To assess the relative importance of Af, mitral regurgitation, plasma BNP (> 77 pg/ml), E/A (> 0.92), LA diameter (> 36 mm), and LAA flow (> 67 cm/s) on cardioembolic stroke classification, we utilized ordinal multivariate logistic regressions. We found that Af, mitral regurgitation, plasma BNP (> 77 pg/ml), and LA dimension (> 36 mm) were statistically significant independent predictors of cardioembolic stroke in the multivariable setting (Χ^2 ^= 127.5, p < 0.001). Odds ratios (OR) and confidence intervals are given in Table 4.

**Table 4:** Odds ratios and 95% confidence intervals for cardioembolic stroke classification, relative to Af, mitral regurgitation, plasma BNP, and LA dimension.

	**n = 131**
**Af**	
No	1.00
Yes	146.3 (19.2, 1115.4)**
**Mitral regurgitation**	
No	1.00
Yes	5.9 (1.2, 28.6)*
**Plasma BNP**	
≤ 77 pg/ml	1.00
> 77 pg/ml	5.1 (1.2, 21.4)*
**LA dimension**	
≤ 36 mm	1.00
> 36 mm	17.3 (3.5, 85.8)**

The multivariate odds ratios are determined from logistic regressions including all four variables (Χ^2 ^= 127.5, p < 0.001). Plasma BNP was independent predictor of cardioembolic stroke, even after controlling Af, mitral regurgitation, and LA dimension.

*: p < 0.05; **: p < 0.01.

## Discussion

In the present study, we evaluated 131 acute ischemic stroke patients, 62 patients with cardioembolic stroke, 69 patients with non-cardioembolic stroke (including atherothrombotic stroke, lacunar stroke, or the other). With these patients, we assessed the prediction factors of plasma BNP and whether we could differentiate among stroke subtypes on the basis of plasma BNP concentrations in addition to patient's clinical variables. Plasma BNP, in acute ischemic stroke patients, was predicted with Af, heart failure, chronic renal failure, and LA diameter. In cardioembolic stroke, the distribution of Af was high, plasma BNP, E/A, and LA diameter were significantly increased, and LAA flow was significantly decreased, compared with non-cardioembolic stroke. Af, mitral regurgitation, plasma BNP (> 77 pg/ml), and LA dimension (> 36 mm) were independent predictors of cardioembolic stroke in the multivariable setting.

BNP at admission was significantly higher in cardioembolic compared with atherothrombotic infarctions [13]. We have found that cardioembolic stroke can be predicted with Af, mitral regurgitation, plasma BNP, and LA diameter. We could differentiate cardioembolic stroke from non-cardioembolic stroke with those non-invasive examination. Recently, Montaner et al. have shown the independent predictors of cardioembolic stroke were atrial fibrillation, other embolic cardiopathies, total anterior circulation infarction, BNP, and D-dimer [14]. Combining their study to our results, plasma BNP can be a surrogate marker of cardioembolic stroke with strong predictive power independent from Af.

It has reported that BNP correlated negatively with LV ejection fraction [15]. In the present study, plasma BNP was also associated with the presence of heart failure or LV ejection fraction in single analysis. However, there is no longer significant association with LV ejection fraction in multivariate analysis. Heart failure covered the significant association of LV ejection fraction with plasma BNP under multivariate setting. On the other hand, plasma BNP following cerebral ischemia was predicted with any LA/LAA abnormality, hemoglobin, chronic heart failure, age, and NIHSS score with 48 patients [16]. In the present study, plasma BNP was strongly associated with LA/LAA abnormality (LA diameter) in 131 patients. It is widely known that stressed LA is one of the cardiac sources to secreting BNP. In their study, age and NIHSS score was also slightly associated with plasma BNP. However, we could not detect any association on plasma BNP with age and NIHSS score. We have found that Af, heart failure, chronic renal failure, and LA diameter, but not age and NIHSS score, predict plasma BNP in acute stroke patients. There are the several reports that supports out results. Plasma BNP is not only reported to be increased in patients with Af [4], cardiomyopathy [5], heart failure [6], myocardial infarction [7], or chronic renal failure [8], but also suggested as related with LA diameter [17].

There are several reports that evaluated the prediction with plasma BNP on the outcome of acute stroke patients. However, it is still controversial whether plasma BNP can predict the outcome of acute stroke patients. High BNP is independent predictor of myocardial infarction and congestive heart failure risk after stroke or transient ischemic attack [18,19]. And, elevated BNP predicted mortality after stroke [20]. High BNP values were associated with death, a first major cardiovascular event, atrial fibrillation, stroke or transient ischemic attack, and heart failure [21]. Proportion of patients with good outcome was significantly reduced in the group with highest BNP quartile [22]. However, using multivariate regression analysis, no significant relation to morbidity and mortality was found for BNP [22]. It has also shown that elevation of BNP in 48 hours after stroke onset is associated with the development of cerebral edema [23].

Handke et al. reported that, independent of the basic rhythm, there was a close relationship between LAA flow and elevated thromboembolic risks in the cerebral ischemia patients [24]. However, to detect LAA flow, TEE has to be done in the very acute state in stroke. It was reported that TEE was identified as a risk factor on pneumonia [25]. Therefore, a more non-invasive marker to predict cardioembolic stroke would be of great interest. From our results, we could differentiate cardioembolic stroke with plasma BNP independent from Af and LA diameter. And, LAA flow was not statistically significant predictor of cardioembolic stroke after controlling Af, plasma BNP, and LA diameter.

For the limitation of this study, the number of patients should be listed. We have evaluated 131 acute stroke patients, and there were 62 cardioembolic stroke patients and 69 non-cardioembolic stroke patients. Investigating larger number of acute stroke patients, exact predictive power of plasma BNP will be detected. Additionally, in our study, we could not detect any patient with patent foramen ovale or pulmonary arteriovenous fistula that are also known to cause cardioembolic stroke. We should also evaluate plasma BNP in those diseases. Secondly, our hospital is high care centered hospital. Therefore, usually heavy symptomatic patients have admitted. And, in the present study, we have evaluated the patients who had admitted within 24 hours after their stroke onset. We think this is the other reason of the high distribution of cardioembolic patients in this study. Hospital based selection bias can be exist.

It is important to differentiate cardioembolic stroke from non-cardioembolic stroke, since cardioembolic stroke generally results in more severe disability and acute treatment and secondary prevention differ in cardioembolic stroke from non-cardioembolic stroke. However, it is difficult to diagnose the subtypes of ischemic stroke accurately at admission. We have evaluated the predictive factors of cardioembolic stroke. From our results, it was suggested that cardioembolic stroke was strongly predicted with Af, plasma BNP, and LA diameter.

## Competing interests

The authors declare that they have no competing interests.

## Authors' contributions

KY conceived of the study, participated in the design of the study, and performed TTE and TEE. NH participated in the stroke subtype diagnosis, statistical analysis, coordination, and drafting the manuscript. TN participated in the design and performed TTE and TEE. TT participated in the stroke subtype diagnosis. HO, MM, and HM participated in the design and performed TTE and TEE. KM and MU performed BNP immunoradiometric assay. HD, TM, and TN participated in the acquisition and statistical analysis of data. YK and MK participated in the design and coordination. All authors read and approved the final manuscript.

## Pre-publication history

The pre-publication history for this paper can be accessed here:


